# Comparison of the UEScope videolaryngoscope with the Macintosh laryngoscope during simulated cardiopulmonary resuscitation: A randomized, cross-over, multi-center manikin study: Erratum

**DOI:** 10.1097/MD.0000000000017125

**Published:** 2019-09-06

In the article, “Comparison of the UEScope videolaryngoscope with the Macintosh laryngoscope during simulated cardiopulmonary resuscitation: A randomized, cross-over, multi-center manikin study”,^[1]^ which appears in Volume 97, Issue 36 of *Medicine*, Dr. Alexander Kaserer's degree should appear as MD, not MD, PhD.
